# Association of bovine lung lesions at slaughter with carcass performance, value and liver-abscess severity

**DOI:** 10.1093/tas/txaf112

**Published:** 2025-08-20

**Authors:** Becca B Grimes, Trenton J McEvers, Travis C Tennant, Ty E Lawrence

**Affiliations:** Beef Carcass Research Center - Department of Agricultural Sciences, West Texas A&M University, Canyon, TX 79016, USA; Beef Carcass Research Center - Department of Agricultural Sciences, West Texas A&M University, Canyon, TX 79016, USA; Beef Carcass Research Center - Department of Agricultural Sciences, West Texas A&M University, Canyon, TX 79016, USA; Beef Carcass Research Center - Department of Agricultural Sciences, West Texas A&M University, Canyon, TX 79016, USA

**Keywords:** beef, carcass quality, liver abscess, lung health

## Abstract

The association of lung abnormalities with carcass performance was evaluated on data from 60,843 carcasses. Lung outcomes were scored for severity of consolidation (N = Normal and < 5% consolidation, 1 = 5 to 15% consolidation, 2 = 15 to 50% consolidation, 3 = > 50% consolidation) and presence of fibrin tags (N = None, M = Minor fibrin, E = Extensive fibrin). Lung consolidation had a strong and detrimental effect (*P* < 0.01) on hot carcass weight, with lung scores of 1, 2, and 3 resulting in 4.2, 13.2, and 29.9 kg less carcass weight compared to carcasses with normal lungs. Minor and extensive fibrin tags, independent of consolidation, also resulted in lighter carcasses (*P* < 0.01; 3.5 kg and 7 kg, respectively). Presence of both lung tissue consolidation and fibrin tags resulted in less (*P *< 0.01) 12th rib fat thickness compared to carcasses with healthy lungs. Similarly, LM area was reduced (*P *< 0.01) in carcasses with lung consolidation (−1.5 to −5.5 cm^2^) or presence of fibrin tags (−2.3 to −2.7 cm^2^) compared to carcasses with healthy lungs. Additionally, severity of lung consolidation and presence of fibrin tags reduced (*P* < 0.01) calculated yield grade values. The greatest proportion of carcasses exhibited edible livers and did not exhibit lung consolidation or fibrin tags (47.67 and 48.88%). A much lower proportion of carcasses (1.12 and 1.89%) exhibited a lung consolidation score of 3 and extensive prevalence of fibrin tags with a major abscess outcome. Within the edible, minor and major abscess category, as lung consolidation increased from normal to 3 and presence of fibrin tags increased from normal to extensive, a decrease in carcass weight (21.5 to 50.1 kg; 5.4 to 7.4 kg), LM area (3.9 to 6.3 cm^2^; 1.6 to 3.1 cm^2^), and 12^th^ rib fat thickness (0.02 to 0.18 cm; 0.12 to 0.30 cm) was observed. Carcasses with an edible liver and lung consolidation scores of 1, 2, and 3, were valued $17.08, $72.27, and $140.59 less than carcasses without lung consolidation based on detriment to carcass weight. This was more pronounced in carcasses exhibiting minor and major liver abscesses in addition to presence of lung consolidation, resulting in -$19.71 to -$222.71 and -$65.70 to -$394.84 less carcass value, respectfully, compared to a carcass with an edible liver and lung. These data indicate that lung and liver health is an important factor that impacts carcass performance and value, particularly carcass weight, muscling and yield grade outcomes.

## Teaser

We evaluated the association of lung and liver health upon carcass performance and value, using a large database of carcasses from the years 2010 to 2021. Lung and liver health were highly associated with carcass performance and value, both of which diminished as lung and liver health worsened.

## INTRODUCTION

Bovine respiratory disease (BRD) is the most challenging and economically devastating disease within the beef industry (NAHMS, 2013; Blakebrough-Hall et al., 2020). Decades of efforts have focused on researching and identifying causative agents (Cravens and Bechtol, 1991; Ellis, 2009), environmental factors (Snowder et al., 2006; Step et al., 2008; Cernicchiaro et al., 2012), treatment protocols (Richeson et al., 2009; Ball et al., 2019; Munoz et al., 2020), and economic losses (Griffin, 1997; Brooks et al., 2011; Johnson and Pendell, 2017; Blakebrough-Hall et al., 2020) of the BRD complex. Even with these efforts, it is apparent that the issue of BRD, and particularly BRD-related death loss, is not improving, and if anything, has worsened over time (NAHMS, 2013; Engler et al., 2014; Vogel et al., 2015). The pathogenesis of BRD is considered multifactorial as it is caused by a combination of viral and bacterial pathogens and can also be influenced by environmental and management factors (Duff and Galyean, 2007; Griffin et al., 2010 ; Galyean et al., 2022). Infection with BRD can occur prior to feedlot arrival and during the beginning portion of the feeding period, therefore negatively influencing entire-feeding period growth performance and efficiency (Edwards, 1996). Clinical signs of BRD can be identified in live cattle including depression, lethargy, gaunt appearance, and nasal discharge (Edwards, 2010). The subjective “pull and treat” method for identifying sick cattle is common practice. However, subclinical populations can be misidentified by feedlot personnel and effects of BRD via lung lesions are not apparent until slaughter (Wittum et al., 1996). Research investigating the effects of BRD on feedlot growth performance and carcass characteristics either use treatment records (Holland et al., 2010; Thomson et al., 2012; Wilson et al., 2017), prevalence of lung lesions at slaughter (Bryant et al., 1996; Schneider et al., 2009; Tennant et al., 2014), or both (Gardner et al., 1999; Blakebrough-Hall et al., 2020) in attempt to identify animals that have been affected by BRD during the feeding period. Schneider et al. (2009) observed presence of lung lesions in 60.6% of cattle that were never treated for BRD, either from being overlooked during feedlot health checks or from BRD exposure prior to feedlot arrival. The ability to score lungs and understand disease development assists the industry with identifying trends in cattle type, management, and realizing the total economic loss of cattle affected by BRD beyond lost feedlot performance and treatment costs.

Liver scoring in beef cattle at slaughter has been utilized for decades and has assisted the industry in understanding causations and trends for liver abscess development and effects on cattle and carcass performance. Liver abscesses, another economically devastating issue to the beef industry, are thought to occur primarily due to increased quantity and duration of consuming highly fermentable feedstuffs, with bouts of ruminal acidosis further contributing to the prevalence of abscesses (Nagaraja and Chengappa, 1998). Increased rates of liver abscesses, particularly severe abscesses (presence of multiple large abscesses, abscess adherence to diaphragm or nearby organs, and an open abscess emitting purulent material or combinations thereof), produce carcasses that are lighter in weight and muscling, and result in increased frequency of railed out carcasses and carcass trimming at slaughter (White and Montgomery, 1985; Brown and Lawrence, 2010; Grimes et al., 2024; Herrick et al., 2024). Although not reported in the literature, it is apparent that both BRD-induced lung damage and liver abscesses are often occurring synergistically in our fed cattle population, with unrealized detriments. To the authors knowledge, there are no published data investigating relationships between adverse lung health and liver abscesses. Furthermore, as the effects of lung health on beef cattle performance has shown to be detrimental in live cattle, the objective of this study was to provide the analysis of a large quantity of lung outcomes in relation to carcass performance in addition to investigating and quantifying the total performance and economic loss caused by two major pathological issues faced by the beef production system.

## MATERIALS AND METHODS

No Institutional Animal Care and Use Committee was necessary due to no live animals being overseen and monitored by the authors prior to harvest for data collection purposes. All live animals were harvested under USDA Food Safety Inspection Service supervision in commercial abattoirs.

### Data Collection Procedures

Data (n = 60,843) were collected as part of the services offered by the Beef Carcass Research Center at West Texas A&M University from 2010 to 2021 at 10 commercial beef abattoirs. Qualification for entry into database included presence of lung health and liver abscess outcomes in addition to carcass outcomes including carcass weight (HCW), calculated yield grade (YG), longissimus muscle area (LM area), 12^th^ rib fat thickness, kidney, pelvic, and heart fat percentage (KPH), and marbling score. An indicator of carcass muscling (RATIO) was calculated as LM area, cm^2^/HCW, kg to standardize carcass muscling per unit of carcass weight. The presence of complete carcass data was not a requirement for entry into the database as some carcass data collection requests did not require all carcass outcomes to be collected. Furthermore, a carcass may be missing outcomes due to the nature in which data is collected in a commercial abattoir-setting (i.e., grading camera did not record a marbling score, but a LM area was still obtained; lung score was recorded but liver score was missed, etc.) or lung presented presence of fibrin tags, but no consolidation or vice versa. Carcasses with missing values (i.e., carcass weight recorded but missing grade data, LM area was not captured but marbling was recorded, liver score not captured but lung score was obtained) were included for analysis. Therefore, the number of observations varied among carcass outcomes (n = 6,332 to 34,663). Lungs were visually evaluated for presence of consolidation and physically palpated for determination of fibrin tags and scored on an individual carcass basis by a trained technician following the procedures of Tennant et al. (2014): Normal (healthy lungs, <5% consolidation and without fibrin tags), Minor (presence of minor fibrin tags), Extensive (presence of extensive fibrin tags), 1 (5% to 15% consolidated lung tissue), 2 (15% to 50% consolidated lung tissue), 3 (> 50% consolidated lung tissue). One technician scored outcomes, and an additional personnel member recorded the raw lung data alongside the technician. To ensure consistency, the same technician scored lung outcomes for an entire customer trial, but technicians may have differed among trials and across years. Training for lung scoring was conducted by the same experienced technician for all scorers and scorers were determined to be qualified after at least three training sessions at a commercial beef abattoir. Raw lung outcome data were recorded by individual carcass (i.e., 1E = lung presented extensive fibrin tags with between 5% to 25% consolidation, M = lung presented minor fibrin tags but no consolidation). However, lung classification (consolidation severity and presence of fibrin tags) were analyzed as independent outcomes and were separated for analysis. Therefore, whereas 60,843 represented the individual carcasses used for analysis, frequency of consolidation (n = 49,561) and fibrin tag severity (n = 47,692) differed due to the separation of the two outcomes as described above. Liver abnormalities were observed and recorded during harvest by a single, trained technician and scored as: edible liver; A- = 1 to 2 small abscesses or inactive scars; A = 1 to 2 large abscesses or multiple small abscesses; A+ = multiple large abscesses; A + AD = liver adhered to diaphragm; A + OP = open liver abscess; A + AD/OP = adhered to diaphragm with an open liver abscess. Liver outcomes were also categorized as minor (A- and A) and major abscesses (A+, A + AD, A + OP, A + AD/OP). Personnel employed by the Beef Carcass Research Center were trained according to the above scoring system by scoring alongside experienced employees on the viscera table at a slaughter abattoir to maintain consistency across scorers. Personnel were considered appropriately trained after at least three-to-four successive supervised audits as deemed by the supervising trainer.

### Statistical Analysis

All analyses were performed using SAS (SAS Inc., NC). Individual carcass (n = 60,843) served as the experimental unit. Frequency of lung consolidation and presence of fibrin tags within liver abscess class was determined via the FREQ procedure. The GLIMMIX procedure was used to determine differences in carcass outcomes within lung consolidation and fibrin tag scores and as well as differences in HCW, LM area and 12^th^ rib fat thickness within lung outcomes and liver abscess outcomes with Kenward-Rogers degrees of freedom approximation. Least squares means were generated and separated using the PDIFF option with a Bonferroni adjustment to control for type I error across multiple comparisons.

## RESULTS AND DISCUSSION

### Lung Health and Carcass Performance

Severity of lung consolidation (Table 1) and presence of fibrin tags (Table 2) had an effect (*P *< 0.01) on carcass weight. Mean carcass weight decreased as lung consolidation increased. Carcasses with lung consolidation scores of 1, 2, and 3 weighed less (*P* < 0.01; −4.2, −13.2, and −29.9 kg) compared to those with normal lungs. Likewise, presence of minor and extensive fibrin tags also resulted in less carcass weight (*P *< 0.01; −3.5 and 7.0 kg) compared to those with normal lungs, a less severe response compared to that of lung consolidation. Tennant et al. (2014) also reported a linear decrease in carcass weight as lung consolidation increased in severity, but reported no difference in carcass weight between carcasses with fibrin tags and those with normal lungs. In agreement with the current study, Bryant et al. (1996) and Gardner et al. (1999) reported losses of 27 to 33 kg in carcass weight in cattle exhibiting lung lesions (lung consolidation) compared to none. Carcasses with presence of lung consolidation (1, 2, and 3 scores) also had smaller (*P *< 0.01) LM area than carcasses with normal lungs (−1.5, −3.8, and −5.5 cm^2^, respectively). Likewise, presence of fibrin tags (minor and extensive) resulted in smaller LM area (−2.7 and −2.3 cm^2^) compared to those with normal lungs. However, no difference (*P *= 0.65) in LM area occurred between minor and extensive fibrin tag scores. Tennant et al. (2014) reported smaller LM area in steers with greater than 50% lung consolidation compared to normal lungs, but observed no difference in LM area among carcasses with normal lungs, fibrin tags, and 5 to 50% lung consolidation. A weight corrected indicator of carcass muscling, RATIO, was affected (*P *< 0.01) by lung health. Between normal and lung consolidation scores of 1 and 2, a 0.0023 and 0.0055 cm^2^/kg decrease (*P *≤ 0.01) in RATIO was observed. In contrast, a lung score of 3 resulted in a numerical increase of 0.0022 cm^2^/kg, but was not different (*P *= 0.52) from normal lungs. These responses can be explained by the difference in trends in HCW and LM area with worsening lung consolidation. Carcass weight decreased at a decreasing rate whereas LM area decreased in a linear manner as lung consolidation worsened. Therefore, cattle with minor to moderate lung consolidation resulted in decreased RATIO, as LM area decreased at a faster rate than HCW. However, in severe lung consolidation, HCW was reduced at a disproportionate rate to LM area, thus inflating RATIO. Carcass RATIO differed (*P *< 0.01) among fibrin tag scores. However, the smallest RATIO (0.2282 cm^2^/kg) occurred in carcasses with minor fibrin tag scores rather than extensive. The authors believe that this result, while statistically significant, may not be biologically relevant as the difference between RATIO in carcasses with minor and extensive fibrin tags is minimal (−0.0027 cm^2^/kg). Moreover, the dissimilar response of lung health on RATIO compared to HCW is likely due to the pronounced reduction in HCW without an equivalent response in LM area.

**Table 1.** Beef carcass yield and quality attributes of carcasses with normal lungs and carcasses with lung consolidation.

		Yield and quality attributes						
Lung Score^1^	n	HCW, kg	12^th^ rib fat thickness, cm	LM area, cm^2^	KPH, %	Yield Grade	Marbling Score^2^	RATIO^3^, cm^2^/kg
Normal	26,994	389.8^a^	1.44^a^	93.1^a^	2.24^a^	3.00^a^	43.4	0.2349^a^
1	10,238	385.6^b^	1.35^b^	91.6^b^	2.05^b^	2.92^b^	43.8	0.2326^b^
2	8,744	376.6^c^	1.23^c^	89.3^c^	2.03^b^	2.84^c^	43.9	0.2294^c^
3	3,585	359.9^d^	1.35^b^	87.6^d^	2.02^b^	2.79^c^	43.9	0.2370^a^
SEM	-	0.46	0.011	0.21	0.008	0.015	0.17	0.00056
*P*-value	-	<0.01	<0.01	<0.01	<0.01	<0.01	0.07	<0.01

^a-d^Means within a column that do not have a common superscript differ (*P* < 0.05).

^1^Normal = No presence of consolidation; 1 = 5 to 15% consolidation; 2 = 15 to 50% consolidation; 3 = > 50% consolidation.

^2^Scores: 30 to 39 = Slight; 40 to 49 = Small; 50 to 59 = Modest.

^3^RATIO = Calculated as LM area, cm^2^/HCW, kg.

**Table 2.** Beef carcass yield and quality attributes of carcasses with normal lungs and carcasses with lungs exhibiting presence of fibrin tags.

		Yield and quality attributes						
Lung Score^1^	n	HCW, kg	12^th^ rib fat thickness, cm	LM area, cm^2^	KPH, %	Yield Grade	Marbling Score^2^	RATIO^3^, cm^2^/kg
Normal	26,994	389.8^a^	1.44^a^	93.1^a^	2.24^a^	3.00^a^	43.4^b^	0.2349^a^
Minor	10,684	386.3^b^	1.26^c^	90.4^b^	2.07^b^	2.90^b^	44.2^a^	0.2282^c^
Extensive	10,014	382.8^c^	1.30^b^	90.8^b^	2.06^b^	2.87^b^	44.1^a^	0.2309^b^
SEM	-	0.41	0.009	0.19	0.007	0.013	0.15	0.00050
*P*-value	-	<0.01	<0.01	< 0.01	<0.01	<0.01	< 0.01	< 0.01

^a-c^Means within a column that do not have a common superscript differ (*P* < 0.05).

^1^Normal = No presence of fibrin; Minor = Minor fibrin; Extensive = Extensive fibrin.

^2^Scores: 30 to 39 = Slight; 40 to 49 = Small; 50 to 59 = Modest.

^3^RATIO = Calculated as LM area, cm^2^/HCW, kg.

Marbling score tended to increase (*P *= 0.07) as lung consolidation increased. Unexpectedly, presence of fibrin tags also resulted (*P *< 0.01) in increased marbling scores, with no difference (*P *= 1.00) in marbling score occurring between minor and extensive fibrin tags. Presence of minor and extensive fibrin tags resulted in a gain in marbling score of 0.81 and 0.74 units. Tennant et al. (2014) reported steers with lung consolidation scores of 50% or greater exhibited lesser marbling scores (Slight^74^ vs Slight^98^) than steers with normal lungs, results not repeated in the current study. The lack of response of lung consolidation on marbling score may be due to the preexisting genetic potential for an animal to reach USDA Choice prior to entry into a feedyard and the timing to which BRD usually occurs. Furthermore, these responses may be indicative of an inflationary effect on marbling with decreasing LMA. Longissimus muscle area decreased as lung health worsened (consolidation and fibrin tags). However, intramuscular fat was not altered by adverse lung health. Thus, marbling score would likely increase or remain unchanged because decreasing LM area with no change in intramuscular fat content would decrease the lean-to-fat appearance in the surface of the LM area for marbling score determination.

Lung consolidation and presence of fibrin tags were associated with less (*P *< 0.01) 12^th^ rib subcutaneous fat thickness. However, increasing severity of lung consolidation and fibrin tags did not result in a linear response. No difference (*P *= 1.00) in 12^th^ rib fat thickness was observed between carcasses with 1 and 3 lung consolidation scores, and a lung consolidation score 2 resulted in the leanest (1.23 cm; *P *< 0.01) 12^th^ rib fat thickness among scores compared to a normal lung. A similar outcome was observed regarding presence of fibrin tags, where carcasses with a minor fibrin tag score resulted in the leanest (1.26 cm; *P *< 0.01) 12^th^ rib fat thickness. These results coincide with those of Tennant et al. (2014) who also did not observe a linear decrease in 12^th^ rib fat thickness with increasing lung consolidation, but did observe a negative effect of lung consolidation on 12^th^ rib fat thickness compared to normal lungs. However, Tennant et al. (2014) observed no difference in 12^th^ rib fat thickness between carcasses with normal lungs and those with presence of fibrin tags. Percentage of estimated kidney, pelvic, and heart fat was affected (*P *< 0.01) by lung consolidation and presence of fibrin tags and was less in carcasses with lung consolidation and presence of fibrin tags compared to normal lungs. No differences (*P ≥ *0.47) among lung consolidation and fibrin tag score severities were observed.

Due to the effects of lung health on HCW, LM area, and 12^th^ rib fat thickness, it is logical to assume YG was affected in a similar manner. Increasing severity of lung consolidation resulted in a linear decrease in YG (*P *< 0.01), with YG decreasing by 0.08, 0.16, and 0.21 units as lung consolidation scores increased from 1 to 3. No difference (*P *= 0.94) in YG was observed between 2 and 3 lung consolidation scores. Similarly, presence of fibrin tags (minor and extensive) also affected (*P *< 0.01) YG, resulting in 0.10 and 0.13 less units of YG compared to carcasses with normal lungs. No difference (*P *= 0.36) in YG was detected between carcasses with minor and extensive fibrin tags.

Similar to the results of the current study, Gardner et al. (1999) reported carcasses from steers without lesions at harvest had greater HCW and increased estimated percentage KPH. However, unlike the present results, they observed no effect of lung lesions on REA, YG, and 12^th^ rib fat thickness and observed increased marbling scores in steers with lung lesions compared to those without. Schneider et al. (2009) also did not observe any effect of lung lesions on carcass outcomes apart from HCW. Gardner et al. (1999) reported that steers without lung lesions at slaughter had heavier final live weights and average daily gains than those with lesions. Increased live weights, and thus increased HCW, is strongly associated with increased intake and therefore this may indicate that cattle affected by lung consolidation after a BRD infection during the backgrounding or receiving phase never regain full feed intake compared to cattle with no lung consolidation (Gifford et al., 2012). Holland et al. (2010) observed a compensatory response in average daily gain during the finishing phase in beef heifers following BRD treatment during the backgrounding phase. However, heifers treated three times and heifers classified as chronically ill, were not able to fully compensate, and thus required an additional 13 to 21 d to regain the loss in live weight.

### Association of Liver Abscess Outcomes and Lung Health

Frequency of lung consolidation and fibrin tags, regardless of liver abscess severity, was 45.53 and 43.4%, respectively (data not shown). Schneider et al. (2009) reported a greater frequency (61.9%) of cattle within their study that had lung lesions present at slaughter. Overall frequency of carcasses within liver abscess class by lung consolidation and presence of fibrin tags are reported in Table 3. Within abscess class by lung consolidation or fibrin tag scores, the greatest percentage of carcasses (47.67 and 48.88%) exhibited edible livers with a normal lung. This agrees with the results of Herrick et al. (2024), who also observed the greatest proportion of carcasses (60.53 and 53.26%) within their study to have edible livers and no lung consolidation or fibrin tags. Frequency of carcasses within the edible liver abscess category decreased as both lung consolidation and presence of fibrin tags increased in severity. Based on these outcomes, only 1.12% of carcasses exhibited a major abscess with a lung score consolidation of 3 (the most severe combination of liver abscess and lung consolidation score), whereas 1.89% of carcasses exhibited a major abscess with an extensive fibrin tag score (the most severe combination of liver abscess and fibrin tag score).

**Table 3.** Frequency of lung consolidation and presence of fibrin tags within liver abscess class.

	Consolidation, %^1^ (n = 43,440)				Fibrin Tags, %^2^ (n = 42,373)		
Abscess Class^3^	Normal	1	2	3	Normal	Minor	Extensive
Edible	47.67	16.78	12.27	4.42	48.88	18.39	16.14
Minor	5.01	2.24	2.09	1.03	5.14	2.56	2.53
Major	2.67	1.80	2.92	1.12	2.74	1.74	1.89

^1^Consolidation: 1 = 5 to 15% consolidation; 2 = 15 to 50% consolidation; 3 = > 50% consolidation.

^2^Fibrin Tags: M or E = presence of minor (M) or extensive (E) fibrin tag formation or interlobular adhesions between lobes.

^3^Edible = liver did not possess abscess at slaughter; Minor = combination of A- and A liver abscesses (A- = 1 or 2 small abscesses or inactive scars, A = 1 to 2 large abscesses or multiple small abscesses); Major = combination of all other liver abscess outcomes (A+ = multiple small abscesses or 1 or more large active abscesses; A + AD = liver adhered to part of the gastrointestinal tract or diaphragm or both; A + OP = ruptured abscesses; A + AD/OP = liver adhered to part of the gastrointestinal tract or diaphragm or both and ruptured abscess).

Mean carcass outcomes were determined by lung consolidation and liver abscess severity and are reported in Table 4. The greatest mean HCW (391.0 kg) was observed in carcasses that exhibited an edible liver and no lung consolidation, not differing (*P *= 0.97) from those with a minor abscess and normal lung or those with an edible liver and 1 consolidation score. Carcasses exhibiting the most severe lung consolidation (3) and liver abscess outcome (major) produced the lightest carcasses (330.9 kg), differing (*P *< 0.01) from all other lung/liver combinations. This outcome resulted in 60.1 kg less HCW compared to carcasses with a normal liver and lung combination. A decrease in HCW was observed within liver abscess severity as lung consolidation score increased, resulting in 21.4, 30.9 and 50.1 kg less HCW within the edible, minor, and major liver abscess categories, respectfully. It is apparent that both presence of lung consolidation and liver abscesses result in significant detriments to carcass weight through impaired metabolic processes. However, both severe liver abscessation and lung consolidation typically results in adherence to nearby tissues and the thoracic cavity, resulting in excess carcass trimming at slaughter, further contributing to reductions in HCW observed in the present study and others (White and Montgomery et al., 1985; Grimes et al., 2024; Herrick et al., 2024).

**Table 4.** Mean HCW, LM area, and 12^th^ rib fat thickness by lung consolidation and liver abscess severity.

	HCW, kg			LM area, cm^2^			12^th^ rib fat thickness, cm		
	Liver Abscess Class^1^								
Lung Consolidation^2^	Edible	Minor	Major	Edible	Minor	Major	Edible	Minor	Major
Normal	391.0^a^	388.0^abc^	381.0^cde^	94.3	93.1	90.4	1.46^a^	1.44^ab^	1.34^abc^
1	388.4^ab^	384.0^bcd^	373.3^efg^	93.4	89.9	89.6	1.39^ab^	1.20^cde^	1.28^abcd^
2	380.0^ed^	374.2^ef^	365.9^gh^	91.7	88.4	86.6	1.33^abc^	1.13^de^	1.01^e^
3	369.6^fg^	357.1^h^	330.9^i^	89.6	89.2	84.1	1.44^ab^	1.26^abcde^	1.21^bcde^
SEM	-	0.73	-	-	0.35	-	-	0.018	-
*P*-value	-	<0.01	-	-	0.13	-	-	<0.01	-

^a-i^Means within each carcass outcome table that do not have a common superscript differ (*P *< 0.05).

^1^Edible = liver did not possess abscess at slaughter; Minor = combination of A- and A liver abscesses (A- = 1 or 2 small abscesses or inactive scars, A = 1 to 2 large abscesses or multiple small abscesses); Major = combination of all other liver abscess outcomes (A+ = multiple small abscesses or 1 or more large active abscesses; A + AD = liver adhered to part of the gastrointestinal tract or diaphragm or both; A + OP = ruptured abscesses; A + AD/OP = liver adhered to part of the gastrointestinal tract or diaphragm or both and ruptured abscess).

^2^Consolidation: 1 = 5 to 15% consolidation; 2 = 15 to 50% consolidation; 3 = > 50% consolidation.

A carcass price was determined using the USDA Market News weighted average formula net price for dressed steers and heifers from the week of May 27^th^, 2024 (LM_CT151; USDA, 2024). This price was used to make assumptions regarding carcass value based on observed differences in carcass weight among lung and liver outcomes. Using the obtained carcass price of $298/45.35 kg, a carcass with a normal and healthy lung/liver combination would return $2,568.76. For carcasses exhibiting an edible liver within lung consolidation scores (1, 2, and 3), total weight-based carcass value decreased by $17.08, $72.27, and $140.59 with increasing severity of lung consolidation compared to normal lungs. For carcasses exhibiting a minor liver abscess and a normal lung or 1, 2, or 3 lung consolidation score, we would expect a decrease in carcass value of $19.71, $45.99, $110.37, and $222.71 with increasing lung consolidation compared to a carcass with a normal lung and liver. The greatest observed loss in estimated carcass value occurred within the major abscess category. As lung consolidation increased in severity from normal to 3, estimated carcass value losses of $65.70, $116.28, and $164.9 occurred, with a major abscess and lung consolidation score of a 3 (the most severe combination) resulting in $394.84 of estimated weight-based carcass value loss compared to a carcass with a normal lung and edible liver.

The combination of liver abscess and lung consolidation score did not produce (*P *= 0.13) a notable change in LM area. However, meaningful biological differences among lung and liver health outcomes were realized. The largest mean LM area (94.3 cm^2^) was observed in carcasses that exhibited an edible liver and no presence of lung consolidation, whereas the smallest mean LM area (84.1 cm^2^) observed across all liver and lung outcomes was observed in carcasses with a major liver abscess and a lung consolidation score of 3. As both lung consolidation and liver abscess severity increased, a numerical reduction in LM area was observed.

Similar to HCW and LM area, the greatest mean 12^th^ rib fat thickness (1.46 cm) was observed in carcasses that exhibited an edible liver and a normal lung. However, reductions in 12^th^ rib fat thickness did not follow a similar pattern as that of HCW and LM area with increasing severity of lung and liver abscess outcomes. As lung consolidation increased in severity from normal to 3 within the edible liver category, a numerical decrease in carcass fatness was observed from normal to 2 (−0.13 cm). However, no difference was observed (*P *≥ 0.14) in 12^th^ rib fat thickness among lung consolidation scores (N—3) within the edible liver category. Within the minor abscess category, 12^th^ rib fat thickness was numerically less than observed within the edible liver category, and did not decrease linearly with increasing lung consolidation severity. However, similar to the edible category, 12^th^ rib fat thickness was numerically less with presence of lung consolidation compared to normal lungs. Within the major abscess category, an even greater effect on 12^th^ rib fat thickness was observed, with a lung score of 2 resulting in the lowest (1.01 cm) mean across all liver and lung outcomes.

Similar to lung consolidation outcomes, within fibrin tag severity outcomes, the greatest mean HCW (391.0 kg) was observed in carcasses with an edible liver and normal lung (Table 5). However, the effect of presence of fibrin tags and liver abscess severity did not affect (*P *= 0.84) HCW. Presence of lung consolidation indicates damage of lung tissue following infection and immune-mediated inflammation (Panciera and Confer, 2010) and undoubtedly impairs oxygen exchange with increasing severity (Baruch et al., 2019), whereas fibrin tags result from the accumulation of interstitial fibrinous connective tissue which is triggered by an inflammatory response following lung tissue damage (Car et al., 1991; Kiser et al., 2017). Therefore, the pronounced effects of lung consolidation on HCW compared to fibrin tags observed is not surprising given that consolidation of lung tissue would greatly impact overall respiratory function and capacity (Pierson and Kainer, 1980; Baruch et al., 2019), whereas fibrin tags primarily affect the interstitial spaces of lung tissue and would not cause serious implications until fibrosis occurred (Kiser et al., 2017).

**Table 5.** Mean HCW, LM area, and 12^th^ rib fat thickness by presence of fibrin tags and liver abscess severity.

	HCW, kg			LMA, cm^2^			12^th^ rib fat thickness, cm		
	Liver Abscess Class^1^								
Fibrin Tags^2^	Edible	Minor	Major	Edible	Minor	Major	Edible	Minor	Major
Normal	391.0	388.0	381.0	94.3	93.1	90.4	1.46^a^	1.44^a^	1.34^ab^
Minor	387.9	385.1	379.9	91.7	87.7	88.3	1.30^b^	1.09^c^	1.02^c^
Extensive	385.5	380.6	375.6	92.0	90.0	88.8	1.34^ab^	1.14^c^	1.10^c^
SEM	-	0.64	-	-	0.30	-	-	0.015	-
*P*-value	-	0.84	-	-	0.07	-	-	<0.01	-

^a-f^Means within each carcass outcome table that do not have a common superscript differ (*P *< 0.05).

^1^Edible = liver did not possess abscess at slaughter; Minor = combination of A- and A liver abscesses (A- = 1 or 2 small abscesses or inactive scars, A = 1 to 2 large abscesses or multiple small abscesses); Major = combination of all other liver abscess outcomes (A+ = multiple small abscesses or 1 or more large active abscesses; A + Adhesion = liver adhered to part of the gastrointestinal tract or diaphragm or both; A + Open = ruptured abscesses; A + Adhesion/Open = liver adhered to part of the gastrointestinal tract or diaphragm or both and ruptured abscess).

^2^Fibrin Tags: M or E = presence of minor (M) or extensive (E) fibrin tag formation or interlobular adhesions between lobes.

The combination of fibrin tags and liver abscess severity tended (*P *= 0.07) to affect LM area. The greatest mean LM area (94.3 cm^2^) was observed in carcasses that exhibited an edible liver and no presence of fibrin tags. A numerical linear decrease in LM area was observed as both prevalence of fibrin tags and liver abscesses increased in severity, with the smallest LM area observed (87.7 cm^2^) in carcasses that exhibited minor presence of fibrin tags and a minor liver abscess, resulting in 6.6 cm^2^ less LM area compared to unaffected liver and lung outcomes. These results indicate that presence of fibrin tags does not greatly influence LM area as compared to HCW.

Presence of fibrin tags and severity of liver abscesses was associated (*P *< 0.01) with detriments to 12^th^ rib fat thickness. The greatest mean 12^th^ rib fat thickness (1.46 cm) was observed in the carcasses that exhibited an edible liver and no presence of fibrin tags. As prevalence of fibrin tags increased in severity from normal to minor within each liver abscess category (edible, minor, and major) a decrease of 0.12, 0.30, and 0.24 cm was observed. Within the edible liver outcome, presence of minor and extensive fibrin tags resulted in less (*P *< 0.01) 12^th^ rib fat thickness compared to a normal lung, but did not differ (*P *= 0.50) between fibrin tag scores. A similar outcome was observed in the minor and major liver abscess classes, whereas 12^th^ rib fat thickness was less (*P *< 0.01) for carcasses that exhibited presence of fibrin tags compared to normal lungs, but no difference (*P *= 1.00) was observed between minor and extensive fibrin tag scores.

Griffin (2014)  reported that lung adhesions were occasionally observed in association with the inflammatory response across the diaphragm due to the effects of a severe (A + AD) liver abscess. More recently, Herrick et al. (2024) reported carcasses with major abscesses had increased rates of lung consolidation and extensive fibrin tag formation than carcasses with edible livers. There is minimal data published evaluating the association of liver health and lung health on carcass outcomes. While effects of lung health and liver health on carcass outcomes have been reported separately across the literature, data in the current study indicate that prevalence of liver abscesses with lung consolidation often happens simultaneously and effects observed from abscesses or consolidation can greatly enhance the effects of the other regarding carcass performance, especially carcass weight. When an animal is fighting an infection, whether that be in the liver in the form of an abscess, or in the lung as consolidation/fibrin tags, energy is partitioned towards immune function rather than growth (Waggoner et al., 2009). Furthermore, the detrimental effects of severe lung consolidation and major abscesses on carcass weight is likely due to a combination of depressed metabolic processes in addition to increased carcass trimming at the slaughter abattoir to remove lung and/or liver adhesion to the thoracic cavity.

Whereas BRD usually affects calves during the beginning stages of the feeding period (Thompson et al., 2006; Babcock et al., 2009), atypical or acute interstitial pneumonia (AIP), also referred to as acute bovine pulmonary emphysema (ABPE), can afflict cattle later in the feeding period (Ayroud et al., 2000; Haydock et al., 2023). This disease also causes abnormalities in the lung tissue, like that of BRD. However, the pathogenesis of AIP differs from BRD in that it is caused by the absorption and subsequent lung metabolism of 3-methylindole, resulting in lung tissue damage and edema and emphysema in the lungs (Haydock et al., 2022). The pathological observations of ABPE mirror those observed in BRD-affected cattle: presence of acute respiratory distress signs and lung lesions. Acute bovine pulmonary edema is typically observed in cattle that have experienced an abrupt change in diet, typically from lower quality forage to high quality forage (Deslandes et al., 2001). Therefore, cases in feedlot cattle are minimal, and often happen sporadically (McAllister, 2013), but an abrupt change in diet can occur during the feeding of fed-beef cattle. Therefore, lung lesions observed at slaughter may not always be caused solely by infection with BRD-associated pathogens.

## CONCLUSION

The full extent of BRD remains unrealized due to misdiagnosis and lack of outward clinical symptoms; evidence of BRD-related illness is often not detected until death or slaughter. The ability to perform lung scoring postmortem has assisted the industry with identifying cattle that were truly affected by BRD-related infections and severity of lung tissue damage, as well as provide information into cattle type, management practices, and overall economic loss. The detrimental effects of BRD on live animal performance has been well-documented, but the results from the current study illustrates that loss in carcass performance is equally pertinent to losses in live performance and are further exacerbated by presence of liver abscesses. The association between liver and lung health has been hypothesized to be a synergistic effect, with detriments in lung health influencing effects of adverse liver abscess conditions and vice versa. Data from the current study indicates that as severity of lung abnormalities and liver abscesses intensify simultaneously, the effects on carcass outcomes are increasingly worsened. These outcomes indicate the effects to which compromised liver and lung function have on carcass outcomes.
